# Calvin G. Goodrich, M.D.

**Published:** 1880-12

**Authors:** 


We clip the following from the Minneapolis *Tribune* of November 21st, 1880:

Dr. Calvin G. Goodrich died at his residence, corner of Seventh Street and Fourth Avenue South, at 11 a. m. yesterday. The above simple announcement, though not a matter of surprise to the many friends of the deceased, is nevertheless a cause for deep regret and wide-spread sorrow. In the death of Dr. Goodrich a wife has lost a noble and generous husband; children will mourn a kind, sympathetic and loving father; the members of the medical profession will part with an honored, intelligent and eminent representative; and Minneapolis will lose a valued, high-minded and useful citizen. Centenary Church of this city has lost an influential member, and God has called home a good and faithful servant.

Dr. Goodrich was born at Petersburg, Va., May 11, 1820, and was one of a family of eleven children. His father, a prominent Virginia lawyer, died when the deceased was but six years old, and an education and a start in life had to be secured as the result of many self-denials, and an indomitable perseverance and personal energy. Entering school at Greencastle, Indiana, where the family afterward resided, Dr. Goodrich finished his education —at the same time contributing to the support of his mother and her family. His next step in life was to enter the Medical College of Cincinnati, Ohio, where he graduated in 1845, and immediately began the practice of his chosen profession at Richmond, Indiana, where he remained until 1848, when he removed to

Oxford, Ohio, where he remained until 1868, the date of his removal to Minneapolis. Since he made this city his home, more than twelve years ago, his daily life has been familiar to all, and does not need to be reviewed. He purchased upon his arrival, of T. A. Harrison, his present homestead, where he has ever since resided, making many friends and building up an extensive practice. As a citizen he has always been public spirited, enterprising and above reproach. In the year 1848, deceased was married to Miss Mary A. Wall, of Richmond, Indiana, the mother of the only surviving children, all but one of whom are residents of Minneapolis. They are: Mrs. Thomas Lowry, Mrs. Volney S. Irish, E. L. Goodrich, and Calvin Goodrich, Jr. Mrs. Goodrich died on the 25th day of the present month, eight years ago, loved and mourned by all who knew her. In 1875 Dr. Goodrich was re-married to Mrs. Harriet Dodman, of Worcester, Mass., who survives her husband, and has the sympathy of friends and the entire community in the deep affliction which has befallen her. Deceased had been ill for several weeks, death finally resulting from pneumonia and quick consumption.

